# Developing the minimum data set of the corrosive ingestion registry system in Iran

**DOI:** 10.1186/s12913-022-08576-0

**Published:** 2022-09-27

**Authors:** Zahra Mahmoudvand, Mostafa Shanbehzadeh, Mohsen Shafiee, Hadi Kazemi-Arpanahi

**Affiliations:** 1 Department of Health Information Technology, School of Allied Medical Sciences, Mazandaran University of Medical Sciences, Mazandaran, Iran; 2 Department of Health Information Technology, School of Paramedical, Ilam University of Medical Sciences, Ilam, Iran; 3 Department of Nursing, Abadan University of Medical Sciences, Abadan, Iran; 4 Department of Health Information Technology, Abadan University of Medical Sciences, Abadan, Iran; 5 Student Research Committee, Abadan University of Medical Sciences, Abadan, Iran

**Keywords:** Registries, Common data elements, Poisoning, Acids, Alkalis, Caustics

## Abstract

**Background:**

Corrosive ingestion is still a major health problem, and its outcomes are often unpredicted. The implementation of a registry system for poisoning with corrosive substances may improve the quality of patient care and might be useful to manage this type of poisoning and its complications. Therefore, our study aimed to establish a minimum data set (MDS) for corrosive ingestion.

**Methods:**

This was an applied study performed in 2022. First, a literature review was conducted to identify the potential data items to be included in the corrosive ingestion MDS. Then, a two-round Delphi survey was performed to attain an agreement among experts regarding the MDS content, and an additional Delphi step was used for confirming the final MDS by calculating the individual item content validity index (CVI) and content validity ratio (CVR) and by using other statistical tests.

**Results:**

After the literature review, 285 data items were collected and sent to a two-round Delphi survey in the form of a questionnaire. In total, 75 experts participated in the Delphi stage, CVI, kappa, and CVR calculation. Finally, the MDS of the corrosive ingestion registry system was identified in two administrative and clinical sections with 21 and 152 data items, respectively.

**Conclusions:**

The development of an MDS, as the first and most important step towards developing the corrosive ingestion registry, can become a standard basis for data collection, reporting, and analysis of corrosive ingestion. We hope this MDS will facilitate epidemiological surveys and assist policymakers by providing higher quality data capture to guide clinical practice and improve patient-centered outcomes.

## Background

Corrosive or caustic ingestion is a serious and life-threatening health problem in clinical toxicology worldwide, especially in low-and middle-income countries (LMICs) [1, 2]. The prevalence ranges from 5,000 to 15,000 cases per year according to national and cultural contexts [3]. Approximately 80% of all cases are children under the age of five years old [4, 5]. Despite accidental ingestion in children, the vast majority of caustic ingestions in adults happen intentionally, i.e., with the intend to commit suicide, and the injuries tend to be more severe [6, 7]. This type of poisoning is a serious problem that often leads to severe complications and even death [8]. Social, economic, and educational factors and mainly the lack of preventative programs contribute to its incidence. However, the incidence of corrosive ingestion and the prevalence of lesions are mostly unreported and unknown. The worldwide epidemiological data are deeply skewed towards well-resourced settings and do not reflect the complete reality of the condition [9]. Similarly, there are no accurate and codified statistics on the epidemiology of poisoning in Iran, including corrosive ingestions [4].

Corrosive substances can be divided into acidic or alkaline chemicals. Substances with a potential of hydrogen (pH) less than two (strong acids) or above 12 (strong alkalis) have a very surface-destructive effect that causes the necrosis of exposed tissues [10]. Ingestions of corrosive substances presents with a wide spectrum of clinical manifestations ranging from asymptomatic to early and severe complications in acute care episodes such as bleeding, esophageal perforation, shock, airway edema, intense pain, and burn in the mouth and back of the chest. Severe complications of corrosive poisoning include chemical damages that occur in the upper part of the gastrointestinal (GI) tract such as inflammatory reaction in the mucosa, esophagitis, and gastritis. Long-term severe complications include stricture of various parts of the GI tract, tracheoesophageal fistula, esophageal shortening, lower esophageal sphincter insufficiency, esophageal dysfunction, intramural false diuretic formation, and esophageal cancer [11–16]. The severity of corrosive ingestion varies depending on several factors, including the nature of the ingested substance, the amount consumed or its concentration, the form (solid or liquid), titratable acid or alkali reserve, duration of GI tissue exposure to the ingested substance, and individual characteristics of the body [17–19].

It has been proven that the occurrence of corrosive damages is growing, especially in LMICs, owing to an absence of effective regulatory and preventive measures and public health surveillance plans. The management of this type of poisoning requires a multidisciplinary and extra-organizational approach as well as effective cooperation between experts in different medical specialties including emergency medicine, surgery, anesthesiology, gastroenterology, ear, nose, and throat (ENT) specialty, and psychiatry. Given the low incidence of corrosive poisoning, caregivers have a limited experience and due to the lack of evidence-based guidelines, there are many ambiguities regarding the best clinical practice. This vagueness is reflected by significant differences in patient management and reported outcomes across the world [20].

In the information age with rising technological advancements, the use of information systems that enhance the ability of health authorities to make informed decisions has become a necessity. Having real data on the events and incidents that lead to injuries and illnesses, as well as the measures taken to help medical staff in both prevention and treatment areas, has a tremendous impact on rescuing patients. Therefore, implementing a poisoning prevention and surveillance program based on longitudinal data collected in a registry system will be a big step toward improving the quality of care and disease control, as well as promoting the health of society as a whole [1]. For this purpose, standard and systematic registration of corrosive ingestion cases is critical for managing poisoning information and treatment strategies and leads to the effective development of poisoning prevention measures [21].

Developing a clinical registry is highly beneficial for organized data collection. The Agency for Healthcare Research and Quality (AHRQ) defines a registry as “a systematized infrastructure that uses observational study methods to collect uniform data for evaluating specified outcomes for a population defined based on a disease, condition, or exposure and that aims to achieve one or more predetermined scientific, clinical, or policy purposes” [22]**.**

Clinical registries are well-constructed tools for tracking and reporting the epidemiological variables of a disease or health condition. These systems can be used to accumulate data on disease progression and patient subgroups, facilitate patient registration into clinical trials, and provide applied evidence regarding the efficiency and cost-effectiveness of new treatments [23]. The poisoning registry system increases the quality of clinical management. This system provides data related to poisonous agents, treatment methods, identification of hazardous agents, and capabilities to manage and analyze the data of poisoned patients. The system is also anticipated to aid in identifying areas and individuals that are possibly at risk of poisoning [24]. However, despite the returns of clinical registries, some requirements should be considered from a data management standpoint, including the design of an effective data collection tool and the identification of the core data elements and their options. Data management in a clinical registry is achieved through several steps, including case discovery, data collection, abstracting and coding, quality control, reporting, and patient follow-up. Case discoveries aim to identify and record all eligible cases. Data collection aims to gather and retain patients’ demographic, therapeutic, follow-up, and historical data in a complete and accurate manner. Abstracting and coding aim to provide a valuable summary of patients’ data. Quality control is a continuing process to ensure the quality of collected data. Reporting is any report published by the registry. Follow-up is performed to monitor patients’ health status after discharge [25, 26]. In this study, we provide a template for the data collection step of the corrosive ingestion registry. Data collection is a laborious process, and it is crucial to capture all data items that are important and relevant to the aim of the registry in order to circumvent the gathering of high-dimension data sets [27].

Minimum data set (MDS) is a standard approach to data collection [28]. It provides a unified template for defining and homogenizing core data elements for a specific disease or clinical condition [29]. It is a set of minimum but adequate data items for collecting data in a standard and agreed manner from a scientific perspective [30]. To collect quality data and achieve an integrated registry system, the existence of an MDS is essential [30, 31]. Registry systems typically use MDS to enable accurate data analysis, decision-making, and appropriate management of poisoning cases [32]. Using an MDS in a clinical registry system is critical to achieve consistent and comparable data on a specific disease or condition [33]. The MDS of corrosive ingestion is required for the ongoing collection and maintenance of the data and is the main requirement for implementing a registry and information system. Since there is no registry system for corrosive ingestion in Iran, the development of an MDS, as the initial phase, is crucial for the collection, processing, analysis, and reporting of proper data to preserve and improve public health. This study aimed to provide an MDS as a template for standardized data collection in the corrosive ingestion registry.

## Methods

In this study, to establish the corrosive ingestion MDS, a multifaceted approach was performed which included the following: First, a literature review was conducted to extract potential variables from scientific and grey (e.g., government health department reports) literature. Then a two-round Delphi stage was performed to select important variables to be included in the corrosive ingestion MDS from experts’ standpoints. Finally, the content of MDS was evaluated using statistical methods. The design and evaluation steps of MDS are as follows:

### Identification of potential data items

A comprehensive systematic review was performed to extract the data items related to corrosive ingestion. First, an extensive search was conducted in scientific databases, such as the Web of Science (WOS), PubMed, ProQuest, Scopus, Science Direct, and Google Scholar, to retrieve the data elements with the potential to be included in the proposed corrosive ingestion MDS. The “advanced search” option was used in PubMed, ProQuest, Google Scholar, and Science Direct and the “document” option was applied in Scopus and WOS.

In this manner, the search formula, which included a combination of search terms, search operators (AND, OR, NOT), and search domains (title, title/abstract, topic), was adjusted to achieve search optimization. The studies were reviewed using the selected keywords including [“Core data element” OR “Core data set” OR “Essential data set” OR “Minimum data set” OR “Minimum data element”] AND [“corrosive” OR “caustic” OR “alkaline” OR “acid*”] AND [“poisoning” OR “intoxication”].

After establishing the search formula, the search criteria were applied through the “search filter” or “refine result” options. Finalized (not in press) full-text journal articles in English with a publication date between January 2000 and April 2022 were included in the study. On the other hand, editorials, books, conference papers, reports, and notes, as well as duplicated, accepted and not finalized, in press, and non-English documents that were published before the year 2000 were excluded. After the adoption of the advanced or document search options and setting the search formula (combining key terms, search operators, and search fields), and applying inclusion and exclusion criteria (via search filter or refine results), the titles and abstracts of potentially relevant studies were independently reviewed by two Health Information Management (HIM) experts (HKA, MSH1). Finally, the full text of any study that mentioned the required data elements of corrosive ingestion was investigated. After that, a data pool of various identified factors was formed. Data elements were extracted from the relevant retrieved resources and entered into a checklist with two administrative and clinical sections.

### Review of potential data items via modified Delphi survey

Providing a valid MDS is the first step in designing clinical registries. To design the MDS, first, an electronic checklist of the extracted factors was developed. The prepared checklist had two main classes and eight subclasses.

In this study, a multidisciplinary panel of 75 experts participated in the Delphi survey and internal content evaluation (CVI, kappa, CVR, and face validity). In the Delphi study, there is no specific method for determining the sample size, but the sample size can be determined based on homogeneity, study time, extension range, availability of specialists, and the study proposed [34, 35]. In this study, we had a homogenous sample of experts including clinicians, researchers, and managers who were involved in the care and treatment of poisoned patients. Therefore, in Delphi studies, when the group of experts is homogeneous, the recommended sample size in different studies is 10–15 individuals, but we identified 75 people based on the available experts to reduce the error rate. To select specialists, the following steps should be considered:

1. First, the related disciplines according to the purpose of the study need to be identified.
2. Specialists in any field must have more than five years of work experience, have an academic degree at a university, and if possible, have publications on poisoning.
3. The answers should be returned to the researchers (if any questionnaire is not returned, the participant will be deleted).

After determining the expert panel, a checklist containing the extracted data items was prepared. Each item represented an important factor in recording or reporting corrosive poisoning. The aim of the study was first explained to the experts by letters and emails, and a consent form for participation in the study was sent to them. Then, an electronic checklist was sent to them via email. The participants were asked to rate each item on a Likert scale ranging from 1 to 5, where 1 indicates insignificant importance and 5 indicates high importance of an item. To add any data items that the experts deemed important, a blank row was provided at the end of the questionnaire. Participants were given a week to rate each item and send the completed checklist back via email. To reduce the error rate, a team of experts blindly evaluated the scores of the items. The evaluation in the Delphi stage is as follows: if less than 60% of the participants agree with the importance of an item, it will be removed. If 60–75% of respondents agree with the importance of that item, it will enter the second phase of Delphi. An item is important if in both the first and second phases of Delphi more than 75% of experts agree with its importance.

### Validity of the content of MDS

After the Delphi phase, important items were extracted and inconsequential items were excluded from the study. Subsequently, the initial MDS template was created and provided to the experts’ panel participating in the Delphi phase to evaluate the content validity. The following steps were taken to assess the content validity:

#### Calculation of content validity index (CVI)

The CVI represents the relevance of each MDS item to the aim of the study and it must be measured for each item. Therefore, the initial MDS was sent to the experts' panel by email, and the experts were asked to rate each item on a 1–4 Likert scale. On this scale, a score of 1 indicated irrelevance and a score of 4 showed the highest level of relevance to the study aim for each item. Specialists were given 15 days to return the initial MDS. To calculate the CVI, the number of specialists who gave the item a score of 3 or 4 is divided by the total number of specialists. The acceptable CVI value is 0.78%. It should be noted that some element of chance is involved in CVI calculation. To eliminate this chance, we also measured S-CVI (universal agreement) and average. It is suggested that a minimum S-CVI of 0.8 reflects content validity.

#### Kappa calculation

As previously mentioned, some element of chance is involved in calculating the CVI. Another method for eliminating this chance is the calculation of kappa. In this study, to eliminate the odds [36], in addition to S-CVI, we also measured kappa for each item using the equation K = (I-CVI-PC)/(1-PC). The interpretation of the kappa statistics for each item is as follows: Kappa values above 0.74 indicate that the element has higher significance to the MDS, values between 0.6 and 0.74 imply acceptable significance, and values between 0.4 and 0.59 indicate that the element has lower significance and should be eliminated from the data set.

#### Calculation of content validity ratio (CVR)

The CVR indicates the necessity of having an item in the MDS form that is relevant to the purpose of the study. In this study, after calculating the CVI, the CVR is determined for each item, and the need for each item is assessed after establishing its importance and relevance. To measure CVR, the filtered MDS from the previous steps is sent to the panel of experts. Experts are asked to rate each item on a Likert scale of 1–3. On this scale, a score of 1 indicates the non-necessity of that item, and a score of 3 indicates the necessity of the item. CVR was calculated using the formula CVR = (Ne—N/2) / (N/2). The participants were given seven days to return the MDS.

#### Calculation of face validity

This criterion evaluates the appearance of the final MDS file and answers the question of whether such a tool is suitable for users. To calculate the face validity for each item, we sent the filtered MDS from the previous steps to the panel of experts. In this study, we evaluated each item in four technical contents: whether the continuity of items is fine, language is understandable, and terminology and given options are simple to understand. The panel of experts was asked to rate each item on a Likert scale of 1–4 in terms of areas of interest. The impact score was calculated using the following formula: Impact score = Frequency (ratio of raters who scored 3 and 4) * Importance (mean score for the importance based on domains). The impact score for each item must be above 1.5, otherwise it will be removed.

## Results

### Systematic review

**Fig. 1:**
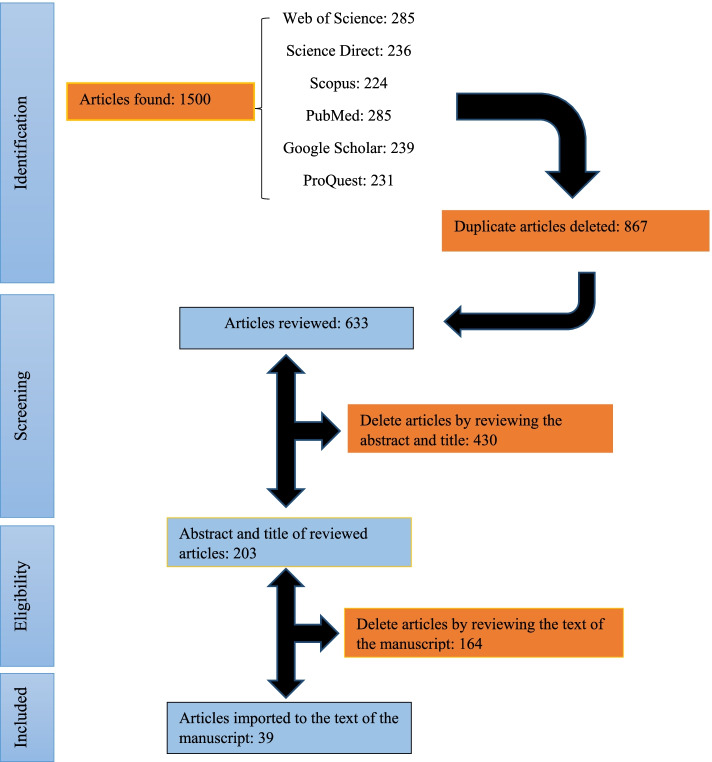
Search flow diagram

In this section, the findings of the systematic review regarding the goal of identifying the required data elements of corrosive ingestion in reviewed databases are presented. In total, 1,500 article titles were searched and entered into EndNote software. After removing the duplicates, 633 papers remained. By applying the inclusion/exclusion criteria and reviewing the titles, 164 articles related to the present study were selected. Then 39 papers were selected to be included in the study after reviewing their abstracts, accessing their full text, and analyzing their content. Our systematic review is reported according to the standard guidelines of the preferred reporting items for systematic reviews and meta-analyses (PRISMA). Figure 1 shows a flowchart of the study selection.

### Review of the national and international poisoning databases

There are several national and international poisoning databases with different contents and structures. We also reviewed existing databases and datasets regarding poisoning (Table 1) until we reached data saturation. Consequently, data items with the potential to be included in the corrosive ingestion MDS were imported to the checklist.

**Table 1:** Poisoning databases/datasets

First author	Country	MDS use case	Data classes	Number of data elements
Robinson et al. [37]	Australia	Poisoning information system	Demographic, exposure factors, signs and symptoms, past medical history, current treatments, and counseling	126
Sabahi et al. [32]	Iran	Poisoning registry system	Clinical section: diagnostic tests, prescription drugs, physical examinations, past medical history,therapeutics procedures, laboratory tests. Administrative section: sociodemographic, lifestyle, contact, and legal	358
Waston et al. [38]	United States	Toxic exposure surveillance system (TESS)	Patient information, exposure information/ substance, clinical impacts (toxic effects), treatment, and medical results	–-
Ekpe et al. [34]	Australia	Record data in hunter area toxicology service (HATS)	Demographics, exposure data, presentation information, past medical history, clinical examinations, psychiatric consultations, treatment plans, patient outcomes, discharge, and follow-up information	88
Yazdipour et al. [39]	Iran	Iranian poisoning registry system	Demographics, patient and communication data, encounter data, diagnostic tests, medical history, exposure data, complications, clinical& treatment plans, paraclinical tests, biobank, and discharge data	558
Whyte et al. [40]	United States	ToxIC registry	Identifiers, encounter data, exposure data, agent data, clinical manifestations, sign and symptoms, and diagnostic& treatment data	48
-[41]	WHO	International toxicology (INTOX) software	Demographics, exposure data, clinical manifestations, referring data, laboratory tests, patient outcomes, and treatment plans	32
Gohari et al. [35]	Iran	Iranian poisoning registry	Clinical section: diagnostic information, exposure information, medical history, clinical findings, diagnostic intervention, and treatment information Non-clinical section: demographic information, presentation information, and discharge information	113
Contini et al. [42]	United States	MDS of the national poison registry system (NPDS)	Socio-demographical, exposure information, clinical findings, patient history, vital signs, physical examinations, diagnostic interventions, complications, clinical syndromes, care plans, follow-up, and medical outcomes	65

### Delphi survey

The expert panel was national in scope and included 12 emergency medicine specialists, 25 toxicologists, two health information management experts, 20 nurses working in the emergency department, two internal medicine specialists, five gastroenterologists, two lung subspecialists, and seven epidemiologists. Moreover, 52% of the panel experts were female with a mean work experience of 18.18 (SD ± 4.5) and a mean age of 45.3 (SD ± 6.3).

A two-round Delphi survey was performed to identify important items of an MDS. In the Delphi phase, a panel of 75 experts was formed. We evaluated the items extracted in the literature review stage in the form of a checklist. The original MDS had two classes, eight subclasses, and 285 items. In the first phase of the Delphi survey, two classes and eight subclasses were approved by a panel of experts and 205 items received a passing score. A total of 75 items were rejected in the first phase of the Delphi survey and five items entered the second phase of Delphi. Of them, three items were accepted by the panel of experts, and two items were rejected. At the end of the Delphi phase, the primary MDS contained 208 important items for experts. Table 2 shows an example of the results of the Delphi survey for two categories.

**Table 2:** An example of the results of the Delphi phases and CVI calculation for two categories (administrative and clinical)

Administrative class									
**Items**	**Delphi phase**						**Calculation of I-CVI**		**Final Decision**
	**Round 1**			**Round 2**					
	**Agree** **N (%)**	**Disagree** **N (%)**	**Unsure** **N (%)**	**Agree** **N (%)**	**Disagree** **N (%)**	**Unsure** **N (%)**	**Relevant** **(Rating 3 or 4)**	**I-CVIs**	
Patients ID	50.66%	24.67%	24.67%				38	0.51	Remove
Age	100	0	0				75	1	Kept
Gender	97.34%	0	2.66%				75	1	Kept
Admission date	76%	20%	4%	80%	16%	4%	70	0.94	Kept
Employment status	33.75%	33.125%	33.125%				42	0.56	Remove
Residence (housing)	92%	5.33%	2.67%				68	0.906	Kept
Income	100	0	0				75	1	Kept
Date of birth	97.33%	2.67%	0				72	0.96	Kept
Marital status	92%	5.33%	2.67%				75	1	Kept
Ethnicity	86.66%	9.33%	4.01%				70	0.933	Kept
Height	90.66%	6.66%	2.68%				69	0.92	Kept
Weight	94.66%	5.34%	0				74	0.986	Kept
National code	46.66%	36.67%	16.67%				35	0.47	Remove
Religion	100%	0	0				68	0.906	Kept
Education level	100%	0	0				67	0.893	Kept
Occupation	100%	0	0				75	1	Kept
Patient's phone number	100%	0	0				30	0.4	Remove
Patient home address	36.66%	46.67%	16.67%				32	0.43	Remove
E-mail	44%	56%	0				33	0.44	Remove
Hospital name	44%	56%	0				33	0.44	Remove
Hospital address	40%	22.5%	37.5%				30	0.4	Remove
Type of referring to the hospital	100%	0	0				75	1	Kept
Dealing with toxin	100%	0	0				74	0.986	Kept
Duration of hospitalization	77.33%	12%	10.67%				75	1	Kept
Admission time	85.33%	10.66%	4.01%				67	0.893	Kept
Patients’ status after discharge	100%	0	0				75	1	Kept
Date of follow-up	100%	0	0				75	1	Kept
Discharge outcome	100%	0	0				75	1	Kept
**Clinical class (Corrosive poisoning subclass)**									
Ingested substance	85.33%	10.66%	4.01%				73	0.973	Kept
Volume of ingestion	77.33%	12%	10.67%				75	1	Kept
Physical characteristics of substances	86.66%	9.33%	4.01%				70	0.933	Kept
Amount of substance	93.33%	6.67%	0				75	1	Kept
Concentration of substance	96%	4%	0%				72	0.96	Kept
Duration time between swallowing until arrival at the hospital	100%	0	0				75	1	Kept
Measures taken before and during transportation to a medical center	60%	26.66%	13.34%	82%	11.33%	6.67%	65	0.866	Kept

### Evaluation of the MDS

#### CVI

The primary MDS items extracted from the Delphi phase were then sent to the expert panel for content assessment. To reduce the odds, we also calculated S-CVI. After important MDS items were evaluated by experts, 15 items were removed and another 193 items were accepted. Following the calculation of CVI and S-CVI, the extracted MDS had 193 items, two classes, and eight subclasses. Table 2 shows an example of the results of the CVI calculation for two categories (administrative class and poisoning subclass).

### Calculation of the kappa value and CVR

To reduce the odds, the kappa value and CVR were calculated for items in each class. Table 2 shows an example of the results of kappa and CVR calculation for each item of two categories (admission class item and poisoning subclass items). After calculating kappa and CVR, experts stated that 12 items of two classes (administrative and clinical) were not necessary and the kappa value for one item was lower than the acceptable value. Therefore, after calculating the kappa and CVR, the prepared MDS had two classes and 181 items.

### Face validity

A total of 181 extracted items were sent from Delphi and CVI phases to an expert panel for face validity review. After calculating face validity, two items were removed and another 173 items were accepted. Table 3 shows the face validity calculation for some items.

**Table 3:** An example of the calculation of CVR, modified kappa, and face validity for two categories (administrative class and corrosive poisoning subclass)

**Data items**	**The number giving a rating of 3 or 4 to the relevancy of the item**	**CVR**^a^	**pc**^b^	**K**^c^	**Face validity**^d^	**Interpretation**
Age	75	1	0/009× ${10}^{-48}$ ~ 0	1	3.5	Excellent
Gender	75	1	~ 0	1	3.5	Excellent
Admission date	70	0.94	~ 0	0.94	3.5	Excellent
Residence (housing)	68	0.906	~ 0	0.906	3.18	Excellent
Income	75	1	~ 0	1	3.5	Excellent
Date of birth	72	0.96	~ 0	0.96	3.5	Excellent
Marital status	75	1	~ 0	1	3.5	Excellent
Ethnicity	70	0.933	~ 0	0.94	2.09	Excellent
Height	69	0.92	~ 0	0.92	3.5	Excellent
Weight	74	0.986	~ 0	0.986	3.01	Excellent
Religion	72	0.96	~ 0	0.96	3.18	Excellent
Education level	75	1	~ 0	1	3.5	Excellent
Occupation	70	0.933	~ 0	0.933	3.04	Excellent
Type of referring to the hospital	69	0.92	~ 0	0.92	3.1	Excellent
Dealing with toxin	75	1	~ 0	1	3.3	Excellent
Duration of hospitalization	74	0.986	~ 0	0.986	3.1	Excellent
Admission time	75	1	~ 0	1	2.89	Excellent
Patients’ status after discharge	67	0.893	~ 0	0.893	3.01	Excellent
Date of follow-up	75	1	~ 0	1	2.94	Excellent
Discharge outcome	75	1	~ 0	1	3.04	Excellent
**Corrosive poisoning subclass**						
Ingested substance	73	0.973	~ 0	0.973	3.3	Excellent
Volume of ingestion	75	1	~ 0	1	3.2	Excellent
Physical characteristics of substances	70	0.933	~ 0	0.933	3.4	Excellent
Amount of substance	75	1	~ 0	1	3.5	Excellent
Concentration of substance	72	0.96	~ 0	0.96	3.5	Excellent
Duration time between swallowing until arrival at the hospital	75	1	~ 0	1	3.5	Excellent
Measures taken before and during transportation to a medical center	65	0.866	~ 0	0.866	3.5	Excellent

^a^The formula of content validity ratio is CVR = (Ne—N/2)/ (N/2). In which the Ne is the number of panelists indicating "essential" and N is the total number of panelists. The numeric value of content validity ratio is determined by Lawshe Table. if CVR is bigger than 0.49, the item in the instrument with an acceptable level of significance will be accepted

^b^Pc (probability of a chance occurrence) was computed using the formula: pc = [N! /A! (N -A)!] *.5Nwhere N = number of experts and A = number of panelists who agree that the item is relevant

^c^K (Modified Kappa) was computed using the formula: K = (I-CVI- PC)/ (1- PC). Interpretation criteria for Kappa, using guidelines described in Cicchetti and Sparrow (1981): Fair = K of 0.40 to 0.59; Good = K of 0.60 to 0.74; and Excellent = K > 0.7

^d^For calculation, the formula Impact Score = frequency (ratio of raters who scored 3 & 4) * Importance (mean score for the importance on the basis of domains) was used. The Impact Score for each item must be above 1.5 or it will be removed

### S-CVI

To eliminate chance and knowledge, the relationship between each of the classes to study S-CVI was examined and Table 4 shows the calculation of S-CVI for the administrative class**.**

**Table 4:** Ratings of the items by 75 experts: items rated 3 or 4 on a 4-point relevance scale (for example)

Data class	Data items	The number of experts	I-CVIs	S-CVI/UA (The proportion of items on a scale that achieves a relevance rating of 3 or 4 by all the experts)	S-CVI/Ave (Average of the I-CVIs for all items on the scale)
Administrative	Age	75	1	S-CVI: 0.92 S-CVI/UA: 0.49	S-CVI/Ave: 0.936
	Gender	75	1		
	Admission date	70	0.94		
	Residence (housing)	68	0.906		
	Income	75	1		
	Date of birth	72	0.96		
	Marital status	75	1		
	Ethnicity	70	0.933		
	Height	69	0.92		
	Weight	74	0.986		
	Religion	72	0.96		
	Education level	75	1		
	Occupation	70	0.933		
	Type of referring to the hospital	69	0.92		
	Dealing with toxin	75	1		
	Duration of hospitalization	74	0.986		
	Admission time	75	1		
	Patients’ status after discharge	67	0.893		
	Date of follow-up	75	1		
	Discharge outcome	75	1		
Clinical (corrosive poisoning subclass)	Ingested substance name	73	0.973	S-CVI: 0.94 S-CVI/UA: 0.48	S-CVI/Ave: 0.948
	Volume of ingestion	75	1		
	Physical characteristics of Substances	70	0.933		
	Amount of substance	75	1		
	Concentration of substance	72	0.96		
	Duration time between ingestion until arrival at the hospital	75	1		
	Measures are taken before and during dispatch to a medical center	65	0.866		

The final MDS had two classes, eight subclasses, and 173 items as follows:

#### Administrative class

This class included two subclasses and 21 items. The subclasses included (1) Socio-demographic and (2) Admission and discharge (Table 5).

**Table 5:** Administrative class

Subclass	Data items
Socio-demographic	Age, gender, residence (housing), income, date of birth, marital status, ethnicity, height, weight, religion, education level, occupation
Admission and discharge (A&D) information	Type of referring to the hospital, dealing with toxin, admission date, admission time, duration of hospitalization, discharge outcome, patients’ status after discharge, date of follow-up

#### Clinical class

This class included six subclasses and 152 items. The subclasses included (1) medical history, (2) poisoning with corrosive substances, (3) clinical manifestations, (4) complications, (5) diagnostic tests, and (6) therapeutic interventions (Table 6).

**Table 6:** Clinical class

Main Category	Sub-class	Data items
Medical history	Drug and substances consumption history	Drug use, alcohol consumption, smoking cigarette
	Comorbidities	Central nervous system (CNS) diseases, cardiovascular diseases (CVD), pulmonary diseases, GI diseases, hepatic disease, renal diseases, skin diseases, muscle-skeletal diseases, hemorrhagic disorders, endocrine disorders, mental disorders
	Family history	Mental disorders, drug consumption
	History of poisoning	Substance name, number of encounters, cause of exposure
Poisoning	Corrosive ingestion	Name of ingested substance, the volume of ingestion, physical characteristics of substances, amount of substance, the concentration of a substance, duration time between ingestion until arrival at the hospital, measures taken before and during transportation to a medical center
Clinical manifestations	CNS manifestations	Coma, delusions, confusion, hallucination, movement disorder, body tremor, myoclonus, level of consciousness, headache, vertigo, convulsions, drowsiness, body temperature changes
	Cardiovascular manifestations	Paroxysmal supraventricular tachycardia (PSVT), extending the QT interval, wide QRS, dysrhythmia, chest pain, blood pressure (BP) disorder, heart rate disorder (tachycardia and bradycardia)
	GI and liver manifestations	Tenderness, diarrhea, abdominal pain, bloody stools, rebound, reflux or gastritis, GI bleeding, liver problems, abdominal rigidity, burned lips, burning of the oral mucosa, taste change in the mouth, tongue edema, pharynx and larynx edema, erythema of the mucous membranes, mouth ulcers, sialorrhea, dysphagia, nausea
	Pulmonary manifestations	Airway edema, respiratory rate changes (tachypnea or brady-pnea), aspiration pneumonia, hoarseness, coughing, stertorous, dyspnea, cyanosis, acute lung injury, chemical pneumonitis, wheezing, acute respiratory distress syndrome (ARDS), hemoptysis, crackle, rales
	Skin and eye manifestations	Facial skin burns, pain at the contact site, burns at the contact site, eczema or rash, angioedema, blister, vesicle, necrosis, abnormal bleeding and bruising, erythema
	Metabolic status	Increased osmotic gap, increased anion gap, hypoglycemia, metabolic acidosis, metabolic alkalosis
Complications		Bleeding, perforation, fistula, esophagus obstruction, mediastinitis, peritonitis, kidney failure, liver dysfunction, hemolysis
Diagnosis tests	Laboratory investigations	Atrial blood gases (ABG), white blood cells (WBC), hemoglobin (hb), hematocrit (HCT), platelet count, retic, lactate, blood sugar (BS), base excess, venus blood gaz (VBG), blood urea nitrogen (BUN), creatinine, sodium, potassium, magnesium, anion gap, troponin, ammonia, creatine kinase (CK), creatine phosphokinase (CPK), prothrombin time (PT), partial thromboplastin time (PTT), international normalize ratio (INR), alanine aminotransferase (ALT), aspartate aminotransferase (AST)
	Para-clinical investigations	Endoscopy, X-ray, CT-scan
Therapeutic interventions	Supportive treatment	Transfusion blood, intravenous (IV) infusion, gavage, airway management, shock management, Oxygen (O2) therapy, esophageal stent
	Prescription medicine	Steroid, antibiotic, antiacid, analgesics
	Type of intervention	Gegeostomy feeding, laparotomy, gastrectomy, esophagectomy, colon interposition, gastric polyp surgery, bougienage

## Discussion

This study aimed to design an MDS for uniform reporting of corrosive ingestion. For this purpose, a comprehensive search in scientific published and grey literature coupled with structured rounds of Delphi survey was performed. The proposed MDS of the corrosive ingestion registry was divided into administrative and clinical sections. The administrative section had two classes, including patients’ demographic data, referral, and follow-up with 21 data items. The clinical section had six main classes, including medical history, poisoning mechanism, clinical findings, complications, diagnostic and treatment measures with 152 data items.

Due to the prevalence and high death rates of poisoning, service centers in the field of poisoning have been established all over the world. These centers provide specialized information to the public and medical staff. These toxicology information centers offer counseling for individuals who have been poisoned and provide education and research services for the prevention and treatment of poisoning [33]. However, based on the searches performed in scientific databases and the review of the systems used by the Ministries of Health of different countries, no coherent information management system was found that specifically covered corrosive ingestion and most of the systems included all poisonings [38, 43, 44].

To develop the corrosive ingestion MDS, the participants were considered to be an illustrative panel in the field of corrosive ingestion on a nationwide level. The Delphi survey is not dependent on a large sample size, but requires a sample size in which agreement and satiety of information can be accomplished. The recommended size of the panel varies from 5 to 20 participants, depending on warranting illustrative verdicts on the target issue [45–48]. The expert panel in our study was 75 experts but included clinicians, managers, and researchers. The suggested level of agreement also differs in the literature from 66%, 75%, or no less than 78% [49]. In this study, we considered 75% as the threshold for agreement and to determine the remaining data items.

Investigating cohesive data sets in a specific field can increase insights and inform the interventions for the inhibition and treatment of illnesses. However, data integration in multi-setting studies is a complex, costly, and slow process. One method to streamline data integration, increase the statistical power of the collected data, integrate results, and reduce costs for academics is to accumulate and report shared data elements [50, 51]. The MDS developed in our study pursues the same purposes to integrate corrosive ingestion data sets and to reply to the inquiries of researchers and clinical trials. Specialists who participated in our study confirmed that the standardization of a corrosive ingestion MDS is appreciated, since it allows for the uniform gathering, analysis, and integration of data. Developing a registry system using our MDS can provide high-quality data, which can assist clinicians and health policymakers in making informed decisions [51].

However, our study has some limitations that must be addressed. First, this study only focused on the informational aspects of the developed MDS, but the technical issues for data interoperability remain to be resolved. Second, given the unknown aspects of corrosive ingestion especially in adults, additional external validation is essential; thus, we recommend conducting a pilot study with a more extensive literature review and consulting with a greater sample of experts, which may improve the MDS. Selecting specialists from a single province is another significant challenge of the study. Therefore, the developed MDS must be evaluated from the standpoint of more multidisciplinary teams all over Iran. Lastly, the Delphi survey was used to reach an agreement on the MDS of corrosive ingestion. This technique has been proven to be fitting for the evaluation of the requirements of health information systems (HISs) [52]. However, by using this method most opinions are disregarded.

The MDS provides a reliable and scientific framework for collecting and reporting corrosive ingestion data. It can enhance the efficiency of the hospitals and clinical settings. The MDS proposed in the present study enables data integration in this field and acts as a basic level for interoperability between HISs. However, it is recommended that upcoming studies consider the technical issues related to interoperability in the corrosive ingestion domain.

## Conclusions

This research offers a fundamental step toward establishing a national registry for corrosive poisoning in Iran from an information management standpoint to improve the data quality criteria. A standardized and agreed-upon MDS is required to provide a more representative picture of corrosive poisoning in Iran. Furthermore, developing a uniform data set helps all the involved parties such as clinicians, police, medicolegal institutes, and policymakers to plan a more appropriate and pervasive plan for the future. An experimental study including a further Delphi step before implementation is advisable to refine some data categories.

## Data Availability

All data generated and analyzed during the current study are not publicly available but are available from the corresponding author upon reasonable request and the Abadan University of Medical Sciences’ approval.

## Abbreviations

MDS: Minimum data set
CVI: Content validity index
CVR: Content validity ratio
LMIC: Low-and middle-income countries
GI: Gastrointestinal
AHRQ: Agency for healthcare research and quality
WOS: Web of Science
HATS: Hunter area toxicology service
TESS: American toxic exposure surveillance system
